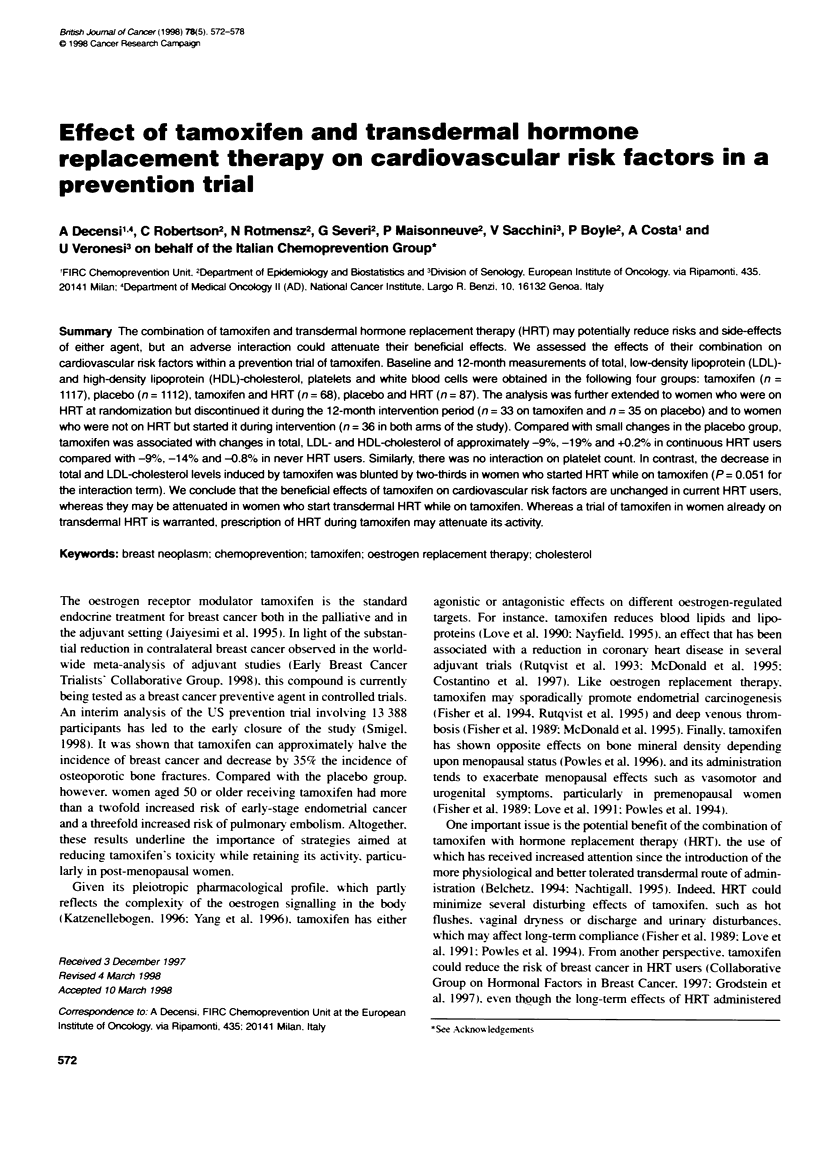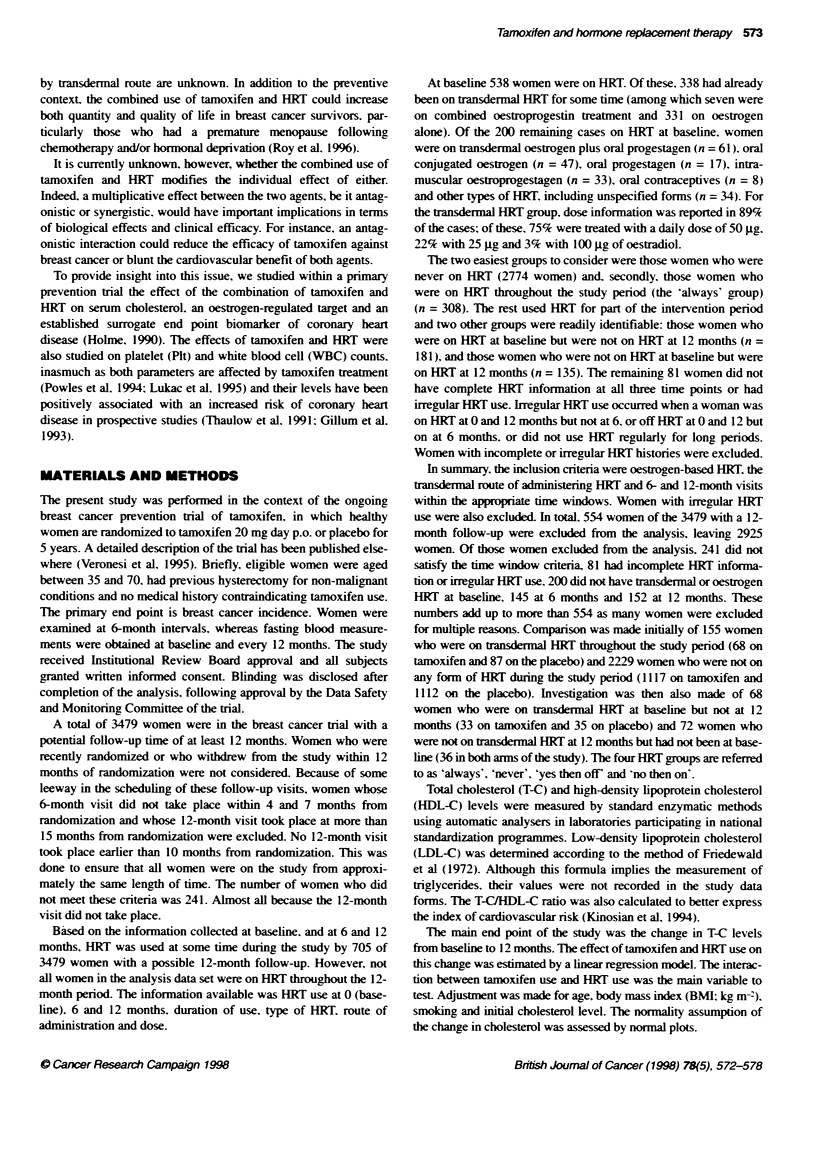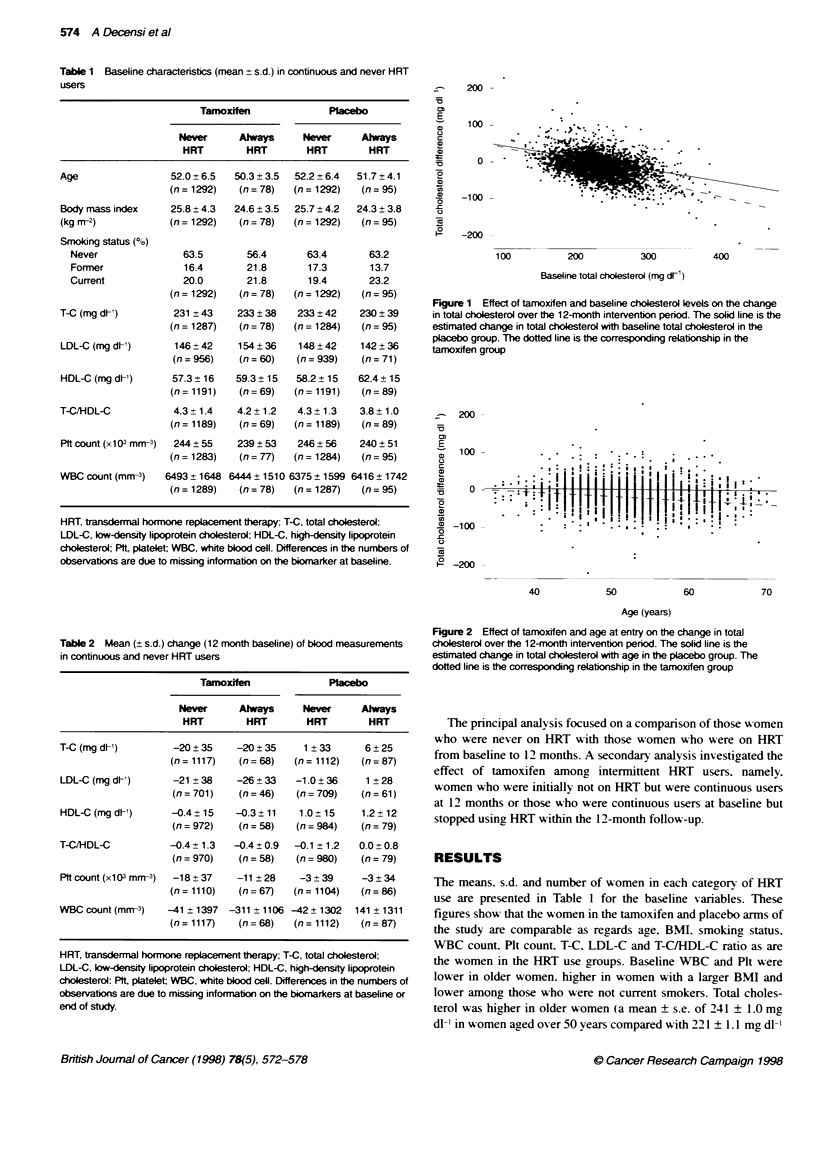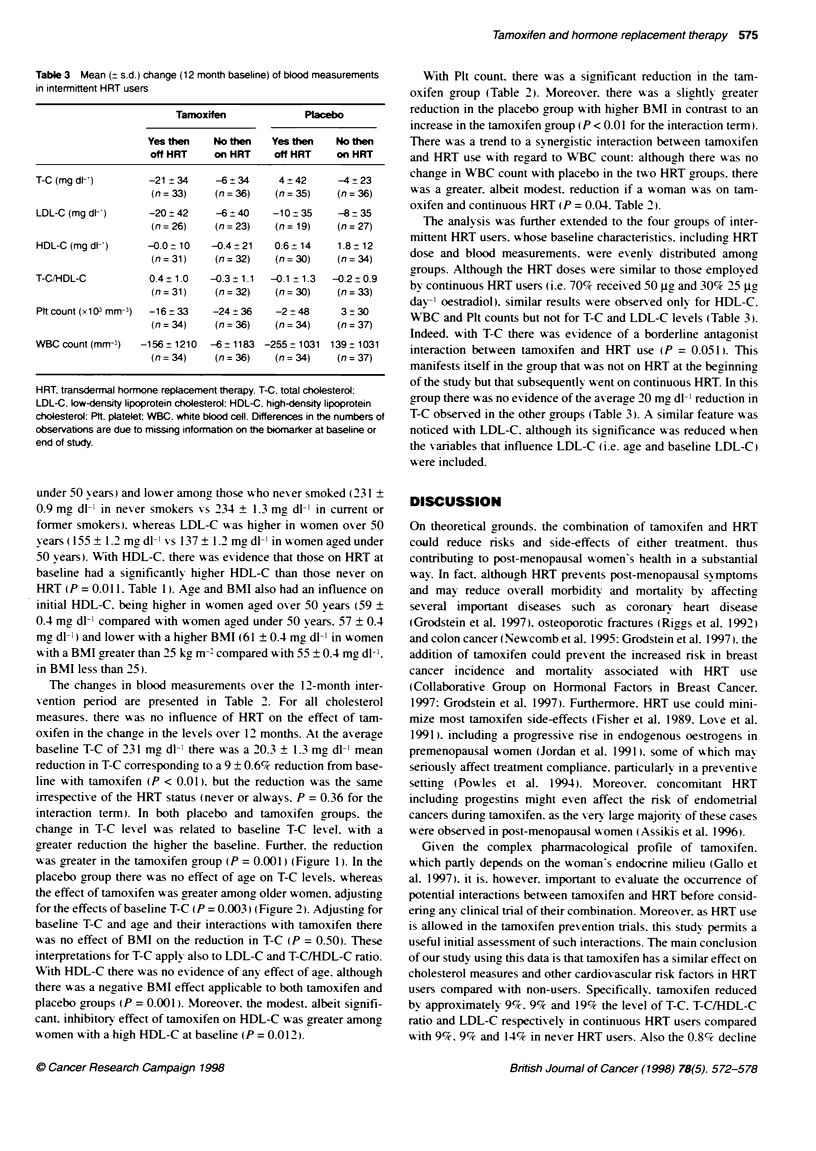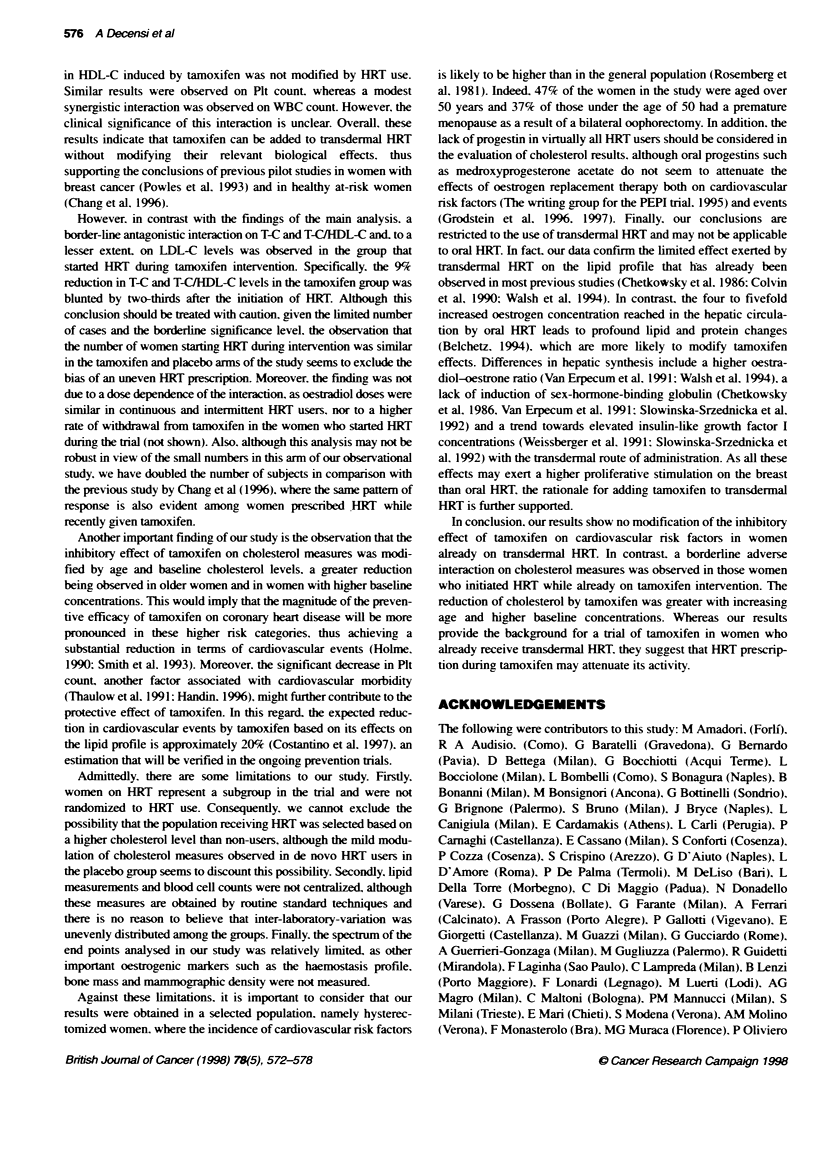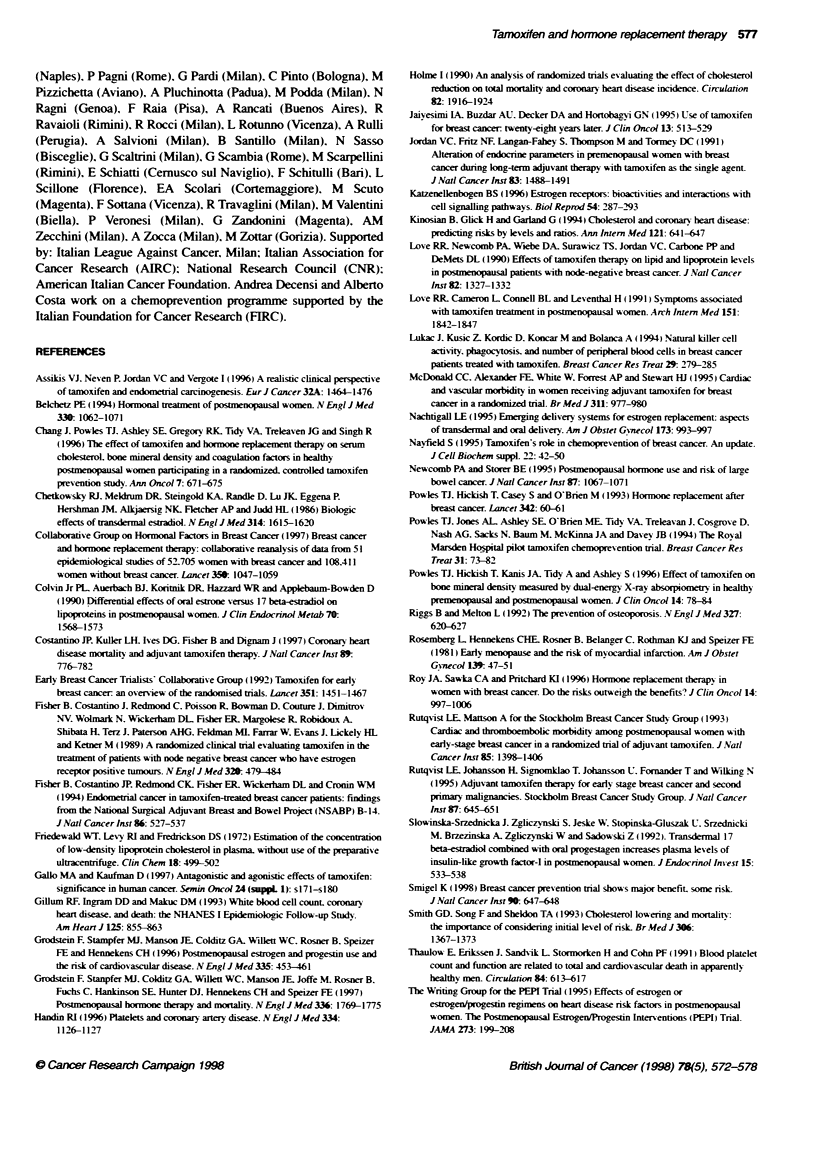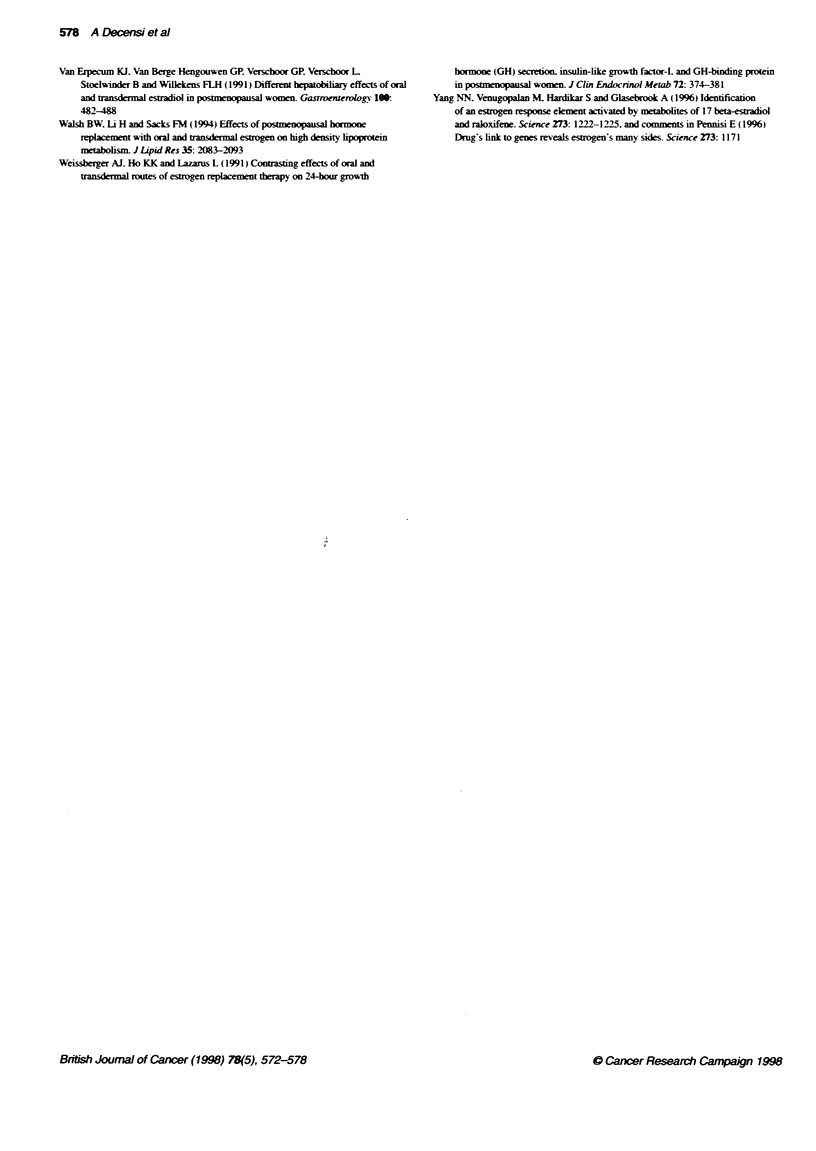# Effect of tamoxifen and transdermal hormone replacement therapy on cardiovascular risk factors in a prevention trial. Italian Chemoprevention Group.

**DOI:** 10.1038/bjc.1998.542

**Published:** 1998-09

**Authors:** A. Decensi, C. Robertson, N. Rotmensz, G. Severi, P. Maisonneuve, V. Sacchini, P. Boyle, A. Costa, U. Veronesi

**Affiliations:** FIRC Chemoprovention Unit, European Institute of Oncology, Milan, Italy.

## Abstract

The combination of tamoxifen and transdermal hormone replacement therapy (HRT) may potentially reduce risks and side-effects of either agent, but an adverse interaction could attenuate their beneficial effects. We assessed the effects of their combination on cardiovascular risk factors within a prevention trial of tamoxifen. Baseline and 12-month measurements of total, low-density lipoprotein (LDL)- and high-density lipoprotein (HDL)-cholesterol, platelets and white blood cells were obtained in the following four groups: tamoxifen (n = 1117), placebo (n = 1112), tamoxifen and HRT (n = 68), placebo and HRT (n = 87). The analysis was further extended to women who were on HRT at randomization but discontinued it during the 12-month intervention period (n = 33 on tamoxifen and n = 35 on placebo) and to women who were not on HRT but started it during intervention (n = 36 in both arms of the study). Compared with small changes in the placebo group, tamoxifen was associated with changes in total, LDL- and HDL-cholesterol of approximately -9%, -19% and +0.2% in continuous HRT users compared with -9%, -14% and -0.8% in never HRT users. Similarly, there was no interaction on platelet count. In contrast, the decrease in total and LDL-cholesterol levels induced by tamoxifen was blunted by two-thirds in women who started HRT while on tamoxifen (P = 0.051 for the interaction term). We conclude that the beneficial effects of tamoxifen on cardiovascular risk factors are unchanged in current HRT users, whereas they may be attenuated in women who start transdermal HRT while on tamoxifen. Whereas a trial of tamoxifen in women already on transdermal HRT is warranted, prescription of HRT during tamoxifen may attenuate its activity.


British Journal of Cancer (1998) 78(5), 572–578
© 1998 Cancer Research Campaign

### Effect of tamoxifen and transdermal hormone replacement therapy on cardiovascular risk factors in a prevention trial

**A Decensi[1,4], C Robertson[2], N Rotmensz[2], G Severi[2], P Maisonneuve[2], V Sacchini[3], P Boyle[2], A Costa[1] and U Veronesi[3] on behalf of the Italian Chemoprevention Group***

[1]FIRC Chemoprevention Unit, [2]Department of Epidemiology and Biostatistics and [3]Division of Senology, European Institute of Oncology, via Ripamonti, 435, 20141 Milan; [4]Department of Medical Oncology II (AD), National Cancer Institute, Largo R. Benzi, 10, 16132 Genoa, Italy

**Summary** The combination of tamoxifen and transdermal hormone replacement therapy (HRT) may potentially reduce risks and side-effects of either agent, but an adverse interaction could attenuate their beneficial effects. We assessed the effects of their combination on cardiovascular risk factors within a prevention trial of tamoxifen. Baseline and 12-month measurements of total, low-density lipoprotein (LDL)- and high-density lipoprotein (HDL)-cholesterol, platelets and white blood cells were obtained in the following four groups: tamoxifen ($n$ = 1117), placebo ($n$ = 1112), tamoxifen and HRT ($n$ = 68), placebo and HRT ($n$ = 87). The analysis was further extended to women who were on HRT at randomization but discontinued it during the 12-month intervention period ($n$ = 33 on tamoxifen and $n$ = 35 on placebo) and to women who were not on HRT but started it during intervention ($n$ = 36 in both arms of the study). Compared with small changes in the placebo group, tamoxifen was associated with changes in total, LDL- and HDL-cholesterol of approximately –9%, –19% and +0.2% in continuous HRT users compared with –9%, –14% and –0.8% in never HRT users. Similarly, there was no interaction on platelet count. In contrast, the decrease in total and LDL-cholesterol levels induced by tamoxifen was blunted by two-thirds in women who started HRT while on tamoxifen ($P$ = 0.051 for the interaction term). We conclude that the beneficial effects of tamoxifen on cardiovascular risk factors are unchanged in current HRT users, whereas they may be attenuated in women who start transdermal HRT while on tamoxifen. Whereas a trial of tamoxifen in women already on transdermal HRT is warranted, prescription of HRT during tamoxifen may attenuate its activity.

**Keywords:** breast neoplasm; chemoprevention; tamoxifen; oestrogen replacement therapy; cholesterol

The oestrogen receptor modulator tamoxifen is the standard endocrine treatment for breast cancer both in the palliative and in the adjuvant setting (Jaiyesimi et al, 1995). In light of the substantial reduction in contralateral breast cancer observed in the worldwide meta-analysis of adjuvant studies (Early Breast Cancer Trialists' Collaborative Group, 1998), this compound is currently being tested as a breast cancer preventive agent in controlled trials. An interim analysis of the US prevention trial involving 13 388 participants has led to the early closure of the study (Smigel, 1998). It was shown that tamoxifen can approximately halve the incidence of breast cancer and decrease by 35% the incidence of osteoporotic bone fractures. Compared with the placebo group, however, women aged 50 or older receiving tamoxifen had more than a twofold increased risk of early-stage endometrial cancer and a threefold increased risk of pulmonary embolism. Altogether, these results underline the importance of strategies aimed at reducing tamoxifen's toxicity while retaining its activity, particularly in post-menopausal women.

Given its pleiotropic pharmacological profile, which partly reflects the complexity of the oestrogen signalling in the body (Katzenellebogen, 1996; Yang et al, 1996), tamoxifen has either agonistic or antagonistic effects on different oestrogen-regulated targets. For instance, tamoxifen reduces blood lipids and lipoproteins (Love et al, 1990; Nayfield, 1995), an effect that has been associated with a reduction in coronary heart disease in several adjuvant trials (Rutqvist et al, 1993; McDonald et al, 1995; Costantino et al, 1997). Like oestrogen replacement therapy, tamoxifen may sporadically promote endometrial carcinogenesis (Fisher et al, 1994, Rutqvist et al, 1995) and deep venous thrombosis (Fisher et al, 1989; McDonald et al, 1995). Finally, tamoxifen has shown opposite effects on bone mineral density depending upon menopausal status (Powles et al, 1996), and its administration tends to exacerbate menopausal effects such as vasomotor and urogenital symptoms, particularly in premenopausal women (Fisher et al, 1989; Love et al, 1991; Powles et al, 1994).

One important issue is the potential benefit of the combination of tamoxifen with hormone replacement therapy (HRT), the use of which has received increased attention since the introduction of the more physiological and better tolerated transdermal route of administration (Belchetz, 1994; Nachtigall, 1995). Indeed, HRT could minimize several disturbing effects of tamoxifen, such as hot flushes, vaginal dryness or discharge and urinary disturbances, which may affect long-term compliance (Fisher et al, 1989; Love et al, 1991; Powles et al, 1994). From another perspective, tamoxifen could reduce the risk of breast cancer in HRT users (Collaborative Group on Hormonal Factors in Breast Cancer, 1997; Grodstein et al, 1997), even though the long-term effects of HRT administered

*Received 3 December 1997*
*Revised 4 March 1998*
*Accepted 10 March 1998*

*Correspondence to:* A Decensi, FIRC Chemoprevention Unit at the European Institute of Oncology, via Ripamonti, 435; 20141 Milan, Italy

*See Acknowledgements by transdermal route are unknown. In addition to the preventive context, the combined use of tamoxifen and HRT could increase both quantity and quality of life in breast cancer survivors, particularly those who had a premature menopause following chemotherapy and/or hormonal deprivation (Roy et al, 1996).

It is currently unknown, however, whether the combined use of tamoxifen and HRT modifies the individual effect of either. Indeed, a multiplicative effect between the two agents, be it antagonistic or synergistic, would have important implications in terms of biological effects and clinical efficacy. For instance, an antagonistic interaction could reduce the efficacy of tamoxifen against breast cancer or blunt the cardiovascular benefit of both agents.

To provide insight into this issue, we studied within a primary prevention trial the effect of the combination of tamoxifen and HRT on serum cholesterol, an oestrogen-regulated target and an established surrogate end point biomarker of coronary heart disease (Holme, 1990). The effects of tamoxifen and HRT were also studied on platelet (Plt) and white blood cell (WBC) counts, inasmuch as both parameters are affected by tamoxifen treatment (Powles et al, 1994; Lukac et al, 1995) and their levels have been positively associated with an increased risk of coronary heart disease in prospective studies (Thaulow et al, 1991; Gillum et al, 1993).

### MATERIALS AND METHODS

The present study was performed in the context of the ongoing breast cancer prevention trial of tamoxifen, in which healthy women are randomized to tamoxifen 20 mg day p.o. or placebo for 5 years. A detailed description of the trial has been published elsewhere (Veronesi et al, 1995). Briefly, eligible women were aged between 35 and 70, had previous hysterectomy for non-malignant conditions and no medical history contraindicating tamoxifen use. The primary end point is breast cancer incidence. Women were examined at 6-month intervals, whereas fasting blood measurements were obtained at baseline and every 12 months. The study received Institutional Review Board approval and all subjects granted written informed consent. Blinding was disclosed after completion of the analysis, following approval by the Data Safety and Monitoring Committee of the trial.

A total of 3479 women were in the breast cancer trial with a potential follow-up time of at least 12 months. Women who were recently randomized or who withdrew from the study within 12 months of randomization were not considered. Because of some leeway in the scheduling of these follow-up visits, women whose 6-month visit did not take place within 4 and 7 months from randomization and whose 12-month visit took place at more than 15 months from randomization were excluded. No 12-month visit took place earlier than 10 months from randomization. This was done to ensure that all women were on the study from approximately the same length of time. The number of women who did not meet these criteria was 241. Almost all because the 12-month visit did not take place.

Based on the information collected at baseline, and at 6 and 12 months, HRT was used at some time during the study by 705 of 3479 women with a possible 12-month follow-up. However, not all women in the analysis data set were on HRT throughout the 12-month period. The information available was HRT use at 0 (baseline), 6 and 12 months, duration of use, type of HRT, route of administration and dose.

At baseline 538 women were on HRT. Of these, 338 had already been on transdermal HRT for some time (among which seven were on combined oestroprogestin treatment and 331 on oestrogen alone). Of the 200 remaining cases on HRT at baseline, women were on transdermal oestrogen plus oral progestagen ($n$ = 61), oral conjugated oestrogen ($n$ = 47), oral progestagen ($n$ = 17), intramuscular oestroprogestagen ($n$ = 33), oral contraceptives ($n$ = 8) and other types of HRT, including unspecified forms ($n$ = 34). For the transdermal HRT group, dose information was reported in 89% of the cases; of these, 75% were treated with a daily dose of 50 μg, 22% with 25 μg and 3% with 100 μg of oestradiol.

The two easiest groups to consider were those women who were never on HRT (2774 women) and, secondly, those women who were on HRT throughout the study period (the 'always' group) ($n$ = 308). The rest used HRT for part of the intervention period and two other groups were readily identifiable: those women who were on HRT at baseline but were not on HRT at 12 months ($n$ = 181), and those women who were not on HRT at baseline but were on HRT at 12 months ($n$ = 135). The remaining 81 women did not have complete HRT information at all three time points or had irregular HRT use. Irregular HRT use occurred when a woman was on HRT at 0 and 12 months but not at 6, or off HRT at 0 and 12 but on at 6 months, or did not use HRT regularly for long periods. Women with incomplete or irregular HRT histories were excluded.

In summary, the inclusion criteria were oestrogen-based HRT, the transdermal route of administering HRT and 6- and 12-month visits within the appropriate time windows. Women with irregular HRT use were also excluded. In total, 554 women of the 3479 with a 12-month follow-up were excluded from the analysis, leaving 2925 women. Of those women excluded from the analysis, 241 did not satisfy the time window criteria, 81 had incomplete HRT information or irregular HRT use, 200 did not have transdermal or oestrogen HRT at baseline, 145 at 6 months and 152 at 12 months. These numbers add up to more than 554 as many women were excluded for multiple reasons. Comparison was made initially of 155 women who were on transdermal HRT throughout the study period (68 on tamoxifen and 87 on the placebo) and 2229 women who were not on any form of HRT during the study period (1117 on tamoxifen and 1112 on the placebo). Investigation was then also made of 68 women who were on transdermal HRT at baseline but not at 12 months (33 on tamoxifen and 35 on placebo) and 72 women who were not on transdermal HRT at 12 months but had not been at baseline (36 in both arms of the study). The four HRT groups are referred to as 'always', 'never', 'yes then off' and 'no then on'.

Total cholesterol (T-C) and high-density lipoprotein cholesterol (HDL-C) levels were measured by standard enzymatic methods using automatic analysers in laboratories participating in national standardization programmes. Low-density lipoprotein cholesterol (LDL-C) was determined according to the method of Friedewald et al (1972). Although this formula implies the measurement of triglycerides, their values were not recorded in the study data forms. The T-C/HDL-C ratio was also calculated to better express the index of cardiovascular risk (Kinosian et al, 1994).

The main end point of the study was the change in T-C levels from baseline to 12 months. The effect of tamoxifen and HRT use on this change was estimated by a linear regression model. The interaction between tamoxifen use and HRT use was the main variable to test. Adjustment was made for age, body mass index (BMI; kg $m^{-2}$), smoking and initial cholesterol level. The normality assumption of the change in cholesterol was assessed by normal plots.

© Cancer Research Campaign 1998

**Table 1** Baseline characteristics (mean ± s.d.) in continuous and never HRT users

| | Tamoxifen | | Placebo | |
|---|---|---|---|---|
| | Never HRT | Always HRT | Never HRT | Always HRT |
| Age | 52.0 ± 6.5 (n = 1292) | 50.3 ± 3.5 (n = 78) | 52.2 ± 6.4 (n = 1292) | 51.7 ± 4.1 (n = 95) |
| Body mass index (kg m$^{-2}$) | 25.8 ± 4.3 (n = 1292) | 24.6 ± 3.5 (n = 78) | 25.7 ± 4.2 (n = 1292) | 24.3 ± 3.8 (n = 95) |
| Smoking status (%) | | | | |
| Never | 63.5 | 56.4 | 63.4 | 63.2 |
| Former | 16.4 | 21.8 | 17.3 | 13.7 |
| Current | 20.0 (n = 1292) | 21.8 (n = 78) | 19.4 (n = 1292) | 23.2 (n = 95) |
| T-C (mg dl$^{-1}$) | 231 ± 43 (n = 1287) | 233 ± 38 (n = 78) | 233 ± 42 (n = 1284) | 230 ± 39 (n = 95) |
| LDL-C (mg dl$^{-1}$) | 146 ± 42 (n = 956) | 154 ± 36 (n = 60) | 148 ± 42 (n = 939) | 142 ± 36 (n = 71) |
| HDL-C (mg dl$^{-1}$) | 57.3 ± 16 (n = 1191) | 59.3 ± 15 (n = 69) | 58.2 ± 15 (n = 1191) | 62.4 ± 15 (n = 89) |
| T-C/HDL-C | 4.3 ± 1.4 (n = 1189) | 4.2 ± 1.2 (n = 69) | 4.3 ± 1.3 (n = 1189) | 3.8 ± 1.0 (n = 89) |
| Plt count (×10$^3$ mm$^{-3}$) | 244 ± 55 (n = 1283) | 239 ± 53 (n = 77) | 246 ± 56 (n = 1284) | 240 ± 51 (n = 95) |
| WBC count (mm$^{-3}$) | 6493 ± 1648 (n = 1289) | 6444 ± 1510 (n = 78) | 6375 ± 1599 (n = 1287) | 6416 ± 1742 (n = 95) |

HRT, transdermal hormone replacement therapy; T-C, total cholesterol; LDL-C, low-density lipoprotein cholesterol; HDL-C, high-density lipoprotein cholesterol; Plt, platelet; WBC, white blood cell. Differences in the numbers of observations are due to missing information on the biomarker at baseline.

**Table 2** Mean (± s.d.) change (12 month baseline) of blood measurements in continuous and never HRT users

| | Tamoxifen | | Placebo | |
|---|---|---|---|---|
| | Never HRT | Always HRT | Never HRT | Always HRT |
| T-C (mg dl$^{-1}$) | −20 ± 35 (n = 1117) | −20 ± 35 (n = 68) | 1 ± 33 (n = 1112) | 6 ± 25 (n = 87) |
| LDL-C (mg dl$^{-1}$) | −21 ± 38 (n = 701) | −26 ± 33 (n = 46) | −1.0 ± 36 (n = 709) | 1 ± 28 (n = 61) |
| HDL-C (mg dl$^{-1}$) | −0.4 ± 15 (n = 972) | −0.3 ± 11 (n = 58) | 1.0 ± 15 (n = 984) | 1.2 ± 12 (n = 79) |
| T-C/HDL-C | −0.4 ± 1.3 (n = 970) | −0.4 ± 0.9 (n = 58) | −0.1 ± 1.2 (n = 980) | 0.0 ± 0.8 (n = 79) |
| Plt count (×10$^3$ mm$^{-3}$) | −18 ± 37 (n = 1110) | −11 ± 28 (n = 67) | −3 ± 39 (n = 1104) | −3 ± 34 (n = 86) |
| WBC count (mm$^{-3}$) | −41 ± 1397 (n = 1117) | −311 ± 1106 (n = 68) | −42 ± 1302 (n = 1112) | 141 ± 1311 (n = 87) |

HRT, transdermal hormone replacement therapy; T-C, total cholesterol; LDL-C, low-density lipoprotein cholesterol; HDL-C, high-density lipoprotein cholesterol: Plt, platelet; WBC, white blood cell. Differences in the numbers of observations are due to missing information on the biomarkers at baseline or end of study.

**Figure 1** Effect of tamoxifen and baseline cholesterol levels on the change in total cholesterol over the 12-month intervention period. The solid line is the estimated change in total cholesterol with baseline total cholesterol in the placebo group. The dotted line is the corresponding relationship in the tamoxifen group

**Figure 2** Effect of tamoxifen and age at entry on the change in total cholesterol over the 12-month intervention period. The solid line is the estimated change in total cholesterol with age in the placebo group. The dotted line is the corresponding relationship in the tamoxifen group

The principal analysis focused on a comparison of those women who were never on HRT with those women who were on HRT from baseline to 12 months. A secondary analysis investigated the effect of tamoxifen among intermittent HRT users, namely, women who were initially not on HRT but were continuous users at 12 months or those who were continuous users at baseline but stopped using HRT within the 12-month follow-up.

### RESULTS

The means, s.d. and number of women in each category of HRT use are presented in Table 1 for the baseline variables. These figures show that the women in the tamoxifen and placebo arms of the study are comparable as regards age, BMI, smoking status, WBC count, Plt count, T-C, LDL-C and T-C/HDL-C ratio as are the women in the HRT use groups. Baseline WBC and Plt were lower in older women, higher in women with a larger BMI and lower among those who were not current smokers. Total cholesterol was higher in older women (a mean ± s.e. of 241 ± 1.0 mg dl$^{-1}$ in women aged over 50 years compared with 221 ± 1.1 mg dl$^{-1}$

© Cancer Research Campaign 1998

**Table 3** Mean (± s.d.) change (12 month baseline) of blood measurements in intermittent HRT users

| | Tamoxifen | | Placebo | |
|---|---|---|---|---|
| | Yes then off HRT | No then on HRT | Yes then off HRT | No then on HRT |
| T-C (mg dl$^{-1}$) | −21 ± 34 ($n$ = 33) | −6 ± 34 ($n$ = 36) | 4 ± 42 ($n$ = 35) | −4 ± 23 ($n$ = 36) |
| LDL-C (mg dl$^{-1}$) | −20 ± 42 ($n$ = 26) | −6 ± 40 ($n$ = 23) | −10 ± 35 ($n$ = 19) | −8 ± 35 ($n$ = 27) |
| HDL-C (mg dl$^{-1}$) | −0.0 ± 10 ($n$ = 31) | −0.4 ± 21 ($n$ = 32) | 0.6 ± 14 ($n$ = 30) | 1.8 ± 12 ($n$ = 34) |
| T-C/HDL-C | 0.4 ± 1.0 ($n$ = 31) | −0.3 ± 1.1 ($n$ = 32) | −0.1 ± 1.3 ($n$ = 30) | −0.2 ± 0.9 ($n$ = 33) |
| Plt count (×10$^3$ mm$^{-3}$) | −16 ± 33 ($n$ = 34) | −24 ± 36 ($n$ = 36) | −2 ± 48 ($n$ = 34) | 3 ± 30 ($n$ = 37) |
| WBC count (mm$^{-3}$) | −156 ± 1210 ($n$ = 34) | −6 ± 1183 ($n$ = 36) | −255 ± 1031 ($n$ = 34) | 139 ± 1031 ($n$ = 37) |

HRT, transdermal hormone replacement therapy. T-C, total cholesterol; LDL-C, low-density lipoprotein cholesterol; HDL-C, high-density lipoprotein cholesterol; Plt, platelet; WBC, white blood cell. Differences in the numbers of observations are due to missing information on the biomarker at baseline or end of study.

under 50 years) and lower among those who never smoked (231 ± 0.9 mg dl$^{-1}$ in never smokers vs 234 ± 1.3 mg dl$^{-1}$ in current or former smokers), whereas LDL-C was higher in women over 50 years (155 ± 1.2 mg dl$^{-1}$ vs 137 ± 1.2 mg dl$^{-1}$ in women aged under 50 years). With HDL-C, there was evidence that those on HRT at baseline had a significantly higher HDL-C than those never on HRT ($P$ = 0.011, Table 1). Age and BMI also had an influence on initial HDL-C, being higher in women aged over 50 years (59 ± 0.4 mg dl$^{-1}$ compared with women aged under 50 years, 57 ± 0.4 mg dl$^{-1}$) and lower with a higher BMI (61 ± 0.4 mg dl$^{-1}$ in women with a BMI greater than 25 kg m$^{-2}$ compared with 55 ± 0.4 mg dl$^{-1}$, in BMI less than 25).

The changes in blood measurements over the 12-month intervention period are presented in Table 2. For all cholesterol measures, there was no influence of HRT on the effect of tamoxifen in the change in the levels over 12 months. At the average baseline T-C of 231 mg dl$^{-1}$ there was a 20.3 ± 1.3 mg dl$^{-1}$ mean reduction in T-C corresponding to a 9 ± 0.6% reduction from baseline with tamoxifen ($P$ < 0.01), but the reduction was the same irrespective of the HRT status (never or always, $P$ = 0.36 for the interaction term). In both placebo and tamoxifen groups, the change in T-C level was related to baseline T-C level, with a greater reduction the higher the baseline. Further, the reduction was greater in the tamoxifen group ($P$ = 0.001) (Figure 1). In the placebo group there was no effect of age on T-C levels, whereas the effect of tamoxifen was greater among older women, adjusting for the effects of baseline T-C ($P$ = 0.003) (Figure 2). Adjusting for baseline T-C and age and their interactions with tamoxifen there was no effect of BMI on the reduction in T-C ($P$ = 0.50). These interpretations for T-C apply also to LDL-C and T-C/HDL-C ratio. With HDL-C there was no evidence of any effect of age, although there was a negative BMI effect applicable to both tamoxifen and placebo groups ($P$ = 0.001). Moreover, the modest, albeit significant, inhibitory effect of tamoxifen on HDL-C was greater among women with a high HDL-C at baseline ($P$ = 0.012).

With Plt count, there was a significant reduction in the tamoxifen group (Table 2). Moreover, there was a slightly greater reduction in the placebo group with higher BMI in contrast to an increase in the tamoxifen group ($P$ < 0.01 for the interaction term). There was a trend to a synergistic interaction between tamoxifen and HRT use with regard to WBC count: although there was no change in WBC count with placebo in the two HRT groups, there was a greater, albeit modest, reduction if a woman was on tamoxifen and continuous HRT ($P$ = 0.04, Table 2).

The analysis was further extended to the four groups of intermittent HRT users, whose baseline characteristics, including HRT dose and blood measurements, were evenly distributed among groups. Although the HRT doses were similar to those employed by continuous HRT users (i.e. 70% received 50 μg and 30% 25 μg day$^{-1}$ oestradiol), similar results were observed only for HDL-C, WBC and Plt counts but not for T-C and LDL-C levels (Table 3). Indeed, with T-C there was evidence of a borderline antagonist interaction between tamoxifen and HRT use ($P$ = 0.051). This manifests itself in the group that was not on HRT at the beginning of the study but that subsequently went on continuous HRT. In this group there was no evidence of the average 20 mg dl$^{-1}$ reduction in T-C observed in the other groups (Table 3). A similar feature was noticed with LDL-C, although its significance was reduced when the variables that influence LDL-C (i.e. age and baseline LDL-C) were included.

### DISCUSSION

On theoretical grounds, the combination of tamoxifen and HRT could reduce risks and side-effects of either treatment, thus contributing to post-menopausal women's health in a substantial way. In fact, although HRT prevents post-menopausal symptoms and may reduce overall morbidity and mortality by affecting several important diseases such as coronary heart disease (Grodstein et al. 1997), osteoporotic fractures (Riggs et al. 1992) and colon cancer (Newcomb et al. 1995; Grodstein et al. 1997), the addition of tamoxifen could prevent the increased risk in breast cancer incidence and mortality associated with HRT use (Collaborative Group on Hormonal Factors in Breast Cancer, 1997; Grodstein et al. 1997). Furthermore, HRT use could minimize most tamoxifen side-effects (Fisher et al. 1989, Love et al. 1991), including a progressive rise in endogenous oestrogens in premenopausal women (Jordan et al. 1991), some of which may seriously affect treatment compliance, particularly in a preventive setting (Powles et al. 1994). Moreover, concomitant HRT including progestins might even affect the risk of endometrial cancers during tamoxifen, as the very large majority of these cases were observed in post-menopausal women (Assikis et al. 1996).

Given the complex pharmacological profile of tamoxifen, which partly depends on the woman's endocrine milieu (Gallo et al. 1997), it is, however, important to evaluate the occurrence of potential interactions between tamoxifen and HRT before considering any clinical trial of their combination. Moreover, as HRT use is allowed in the tamoxifen prevention trials, this study permits a useful initial assessment of such interactions. The main conclusion of our study using this data is that tamoxifen has a similar effect on cholesterol measures and other cardiovascular risk factors in HRT users compared with non-users. Specifically, tamoxifen reduced by approximately 9%, 9% and 19% the level of T-C, T-C/HDL-C ratio and LDL-C respectively in continuous HRT users compared with 9%, 9% and 14% in never HRT users. Also the 0.8% decline

© Cancer Research Campaign 1998

in HDL-C induced by tamoxifen was not modified by HRT use. Similar results were observed on Plt count, whereas a modest synergistic interaction was observed on WBC count. However, the clinical significance of this interaction is unclear. Overall, these results indicate that tamoxifen can be added to transdermal HRT without modifying their relevant biological effects, thus supporting the conclusions of previous pilot studies in women with breast cancer (Powles et al. 1993) and in healthy at-risk women (Chang et al. 1996).

However, in contrast with the findings of the main analysis, a border-line antagonistic interaction on T-C and T-C/HDL-C and, to a lesser extent, on LDL-C levels was observed in the group that started HRT during tamoxifen intervention. Specifically, the 9% reduction in T-C and T-C/HDL-C levels in the tamoxifen group was blunted by two-thirds after the initiation of HRT. Although this conclusion should be treated with caution, given the limited number of cases and the borderline significance level, the observation that the number of women starting HRT during intervention was similar in the tamoxifen and placebo arms of the study seems to exclude the bias of an uneven HRT prescription. Moreover, the finding was not due to a dose dependence of the interaction, as oestradiol doses were similar in continuous and intermittent HRT users, nor to a higher rate of withdrawal from tamoxifen in the women who started HRT during the trial (not shown). Also, although this analysis may not be robust in view of the small numbers in this arm of our observational study, we have doubled the number of subjects in comparison with the previous study by Chang et al (1996), where the same pattern of response is also evident among women prescribed HRT while recently given tamoxifen.

Another important finding of our study is the observation that the inhibitory effect of tamoxifen on cholesterol measures was modified by age and baseline cholesterol levels, a greater reduction being observed in older women and in women with higher baseline concentrations. This would imply that the magnitude of the preventive efficacy of tamoxifen on coronary heart disease will be more pronounced in these higher risk categories, thus achieving a substantial reduction in terms of cardiovascular events (Holme, 1990; Smith et al. 1993). Moreover, the significant decrease in Plt count, another factor associated with cardiovascular morbidity (Thaulow et al. 1991; Handin, 1996), might further contribute to the protective effect of tamoxifen. In this regard, the expected reduction in cardiovascular events by tamoxifen based on its effects on the lipid profile is approximately 20% (Costantino et al. 1997), an estimation that will be verified in the ongoing prevention trials.

Admittedly, there are some limitations to our study. Firstly, women on HRT represent a subgroup in the trial and were not randomized to HRT use. Consequently, we cannot exclude the possibility that the population receiving HRT was selected based on a higher cholesterol level than non-users, although the mild modulation of cholesterol measures observed in de novo HRT users in the placebo group seems to discount this possibility. Secondly, lipid measurements and blood cell counts were not centralized, although these measures are obtained by routine standard techniques and there is no reason to believe that inter-laboratory-variation was unevenly distributed among the groups. Finally, the spectrum of the end points analysed in our study was relatively limited, as other important oestrogenic markers such as the haemostasis profile, bone mass and mammographic density were not measured.

Against these limitations, it is important to consider that our results were obtained in a selected population, namely hysterectomized women, where the incidence of cardiovascular risk factors is likely to be higher than in the general population (Rosemberg et al. 1981). Indeed, 47% of the women in the study were aged over 50 years and 37% of those under the age of 50 had a premature menopause as a result of a bilateral oophorectomy. In addition, the lack of progestin in virtually all HRT users should be considered in the evaluation of cholesterol results, although oral progestins such as medroxyprogesterone acetate do not seem to attenuate the effects of oestrogen replacement therapy both on cardiovascular risk factors (The writing group for the PEPI trial, 1995) and events (Grodstein et al. 1996, 1997). Finally, our conclusions are restricted to the use of transdermal HRT and may not be applicable to oral HRT. In fact, our data confirm the limited effect exerted by transdermal HRT on the lipid profile that has already been observed in most previous studies (Chetkowsky et al. 1986; Colvin et al. 1990; Walsh et al. 1994). In contrast, the four to fivefold increased oestrogen concentration reached in the hepatic circulation by oral HRT leads to profound lipid and protein changes (Belchetz, 1994), which are more likely to modify tamoxifen effects. Differences in hepatic synthesis include a higher oestradiol–oestrone ratio (Van Erpecum et al. 1991; Walsh et al. 1994), a lack of induction of sex-hormone-binding globulin (Chetkowsky et al. 1986, Van Erpecum et al. 1991; Slowinska-Srzednicka et al. 1992) and a trend towards elevated insulin-like growth factor I concentrations (Weissberger et al. 1991; Slowinska-Srzednicka et al. 1992) with the transdermal route of administration. As all these effects may exert a higher proliferative stimulation on the breast than oral HRT, the rationale for adding tamoxifen to transdermal HRT is further supported.

In conclusion, our results show no modification of the inhibitory effect of tamoxifen on cardiovascular risk factors in women already on transdermal HRT. In contrast, a borderline adverse interaction on cholesterol measures was observed in those women who initiated HRT while already on tamoxifen intervention. The reduction of cholesterol by tamoxifen was greater with increasing age and higher baseline concentrations. Whereas our results provide the background for a trial of tamoxifen in women who already receive transdermal HRT, they suggest that HRT prescription during tamoxifen may attenuate its activity.

### ACKNOWLEDGEMENTS

The following were contributors to this study: M Amadori, (Forlí), R A Audisio, (Como), G Baratelli (Gravedona), G Bernardo (Pavia), D Bettega (Milan), G Bocchiotti (Acqui Terme), L Bocciolone (Milan), L Bombelli (Como), S Bonagura (Naples), B Bonanni (Milan), M Bonsignori (Ancona), G Bottinelli (Sondrio), G Brignone (Palermo), S Bruno (Milan), J Bryce (Naples), L Canigiula (Milan), E Cardamakis (Athens), L Carli (Perugia), P Carnaghi (Castellanza), E Cassano (Milan), S Conforti (Cosenza), P Cozza (Cosenza), S Crispino (Arezzo), G D'Aiuto (Naples), L D'Amore (Roma), P De Palma (Termoli), M DeLiso (Bari), L Della Torre (Morbegno), C Di Maggio (Padua), N Donadello (Varese), G Dossena (Bollate), G Farante (Milan), A Ferrari (Calcinato), A Frasson (Porto Alegre), P Gallotti (Vigevano), E Giorgetti (Castellanza), M Guazzi (Milan), G Gucciardo (Rome), A Guerrieri-Gonzaga (Milan), M Gugliuzza (Palermo), R Guidetti (Mirandola), F Laginha (Sao Paulo), C Lampreda (Milan), B Lenzi (Porto Maggiore), F Lonardi (Legnago), M Luerti (Lodi), AG Magro (Milan), C Maltoni (Bologna), PM Mannucci (Milan), S Milani (Trieste), E Mari (Chieti), S Modena (Verona), AM Molino (Verona), F Monasterolo (Bra), MG Muraca (Florence), P Oliviero

 © Cancer Research Campaign 1998

(Naples), P Pagni (Rome), G Pardi (Milan), C Pinto (Bologna), M Pizzichetta (Aviano), A Pluchinotta (Padua), M Podda (Milan), N Ragni (Genoa), F Raia (Pisa), A Rancati (Buenos Aires), R Ravaioli (Rimini), R Rocci (Milan), L Rotunno (Vicenza), A Rulli (Perugia), A Salvioni (Milan), B Santillo (Milan), N Sasso (Bisceglie), G Scaltrini (Milan), G Scambia (Rome), M Scarpellini (Rimini), E Schiatti (Cernusco sul Naviglio), F Schitulli (Bari), L Scillone (Florence), EA Scolari (Cortemaggiore), M Scuto (Magenta), F Sottana (Vicenza), R Travaglini (Milan), M Valentini (Biella), P Veronesi (Milan), G Zandonini (Magenta), AM Zecchini (Milan), A Zocca (Milan), M Zottar (Gorizia). Supported by: Italian League Against Cancer, Milan; Italian Association for Cancer Research (AIRC); National Research Council (CNR); American Italian Cancer Foundation. Andrea Decensi and Alberto Costa work on a chemoprevention programme supported by the Italian Foundation for Cancer Research (FIRC).

### REFERENCES

Assikis VJ, Neven P, Jordan VC and Vergote I (1996) A realistic clinical perspective of tamoxifen and endometrial carcinogenesis. *Eur J Cancer* **32A**: 1464–1476

Belchetz PE (1994) Hormonal treatment of postmenopausal women. *N Engl J Med* **330**: 1062–1071

Chang J, Powles TJ, Ashley SE, Gregory RK, Tidy VA, Treleaven JG and Singh R (1996) The effect of tamoxifen and hormone replacement therapy on serum cholesterol, bone mineral density and coagulation factors in healthy postmenopausal women participating in a randomized, controlled tamoxifen prevention study. *Ann Oncol* **7**: 671–675

Chetkowsky RJ, Meldrum DR, Steingold KA, Randle D, Lu JK, Eggena P, Hershman JM, Alkjaersig NK, Fletcher AP and Judd HL (1986) Biologic effects of transdermal estradiol. *N Engl J Med* **314**: 1615–1620

Collaborative Group on Hormonal Factors in Breast Cancer (1997) Breast cancer and hormone replacement therapy: collaborative reanalysis of data from 51 epidemiological studies of 52,705 women with breast cancer and 108,411 women without breast cancer. *Lancet* **350**: 1047–1059

Colvin Jr PL, Auerbach BJ, Koritnik DR, Hazzard WR and Applebaum-Bowden D (1990) Differential effects of oral estrone versus 17 beta-estradiol on lipoproteins in postmenopausal women. *J Clin Endocrinol Metab* **70**: 1568–1573

Costantino JP, Kuller LH, Ives DG, Fisher B and Dignam J (1997) Coronary heart disease mortality and adjuvant tamoxifen therapy. *J Natl Cancer Inst* **89**: 776–782

Early Breast Cancer Trialists' Collaborative Group (1992) Tamoxifen for early breast cancer: an overview of the randomised trials. *Lancet* **351**: 1451–1467

Fisher B, Costantino J, Redmond C, Poisson R, Bowman D, Couture J, Dimitrov NV, Wolmark N, Wickerham DL, Fisher ER, Margolese R, Robidoux A, Shibata H, Terz J, Paterson AHG, Feldman MI, Farrar W, Evans J, Lickely HL and Ketner M (1989) A randomized clinical trial evaluating tamoxifen in the treatment of patients with node negative breast cancer who have estrogen receptor positive tumours. *N Engl J Med* **320**: 479–484

Fisher B, Costantino JP, Redmond CK, Fisher ER, Wickerham DL and Cronin WM (1994) Endometrial cancer in tamoxifen-treated breast cancer patients: findings from the National Surgical Adjuvant Breast and Bowel Project (NSABP) B-14. *J Natl Cancer Inst* **86**: 527–537

Friedewald WT, Levy RI and Fredrickson DS (1972) Estimation of the concentration of low-density lipoprotein cholesterol in plasma, without use of the preparative ultracentrifuge. *Clin Chem* **18**: 499–502

Gallo MA and Kaufman D (1997) Antagonistic and agonistic effects of tamoxifen: significance in human cancer. *Semin Oncol* **24 (suppl. 1)**: s171–s180

Gillum RF, Ingram DD and Makuc DM (1993) White blood cell count, coronary heart disease, and death: the NHANES I Epidemiologic Follow-up Study. *Am Heart J* **125**: 855–863

Grodstein F, Stampfer MJ, Manson JE, Colditz GA, Willett WC, Rosner B, Speizer FE and Hennekens CH (1996) Postmenopausal estrogen and progestin use and the risk of cardiovascular disease. *N Engl J Med* **335**: 453–461

Grodstein F, Stanpfer MJ, Colditz GA, Willett WC, Manson JE, Joffe M, Rosner B, Fuchs C, Hankinson SE, Hunter DJ, Hennekens CH and Speizer FE (1997) Postmenopausal hormone therapy and mortality. *N Engl J Med* **336**: 1769–1775

Handin RI (1996) Platelets and coronary artery disease. *N Engl J Med* **334**: 1126–1127

Holme I (1990) An analysis of randomized trials evaluating the effect of cholesterol reduction on total mortality and coronary heart disease incidence. *Circulation* **82**: 1916–1924

Jaiyesimi IA, Buzdar AU, Decker DA and Hortobagyi GN (1995) Use of tamoxifen for breast cancer: twenty-eight years later. *J Clin Oncol* **13**: 513–529

Jordan VC, Fritz NF, Langan-Fahey S, Thompson M and Tormey DC (1991) Alteration of endocrine parameters in premenopausal women with breast cancer during long-term adjuvant therapy with tamoxifen as the single agent. *J Natl Cancer Inst* **83**: 1488–1491

Katzenellenbogen BS (1996) Estrogen receptors: bioactivities and interactions with cell signalling pathways. *Biol Reprod* **54**: 287–293

Kinosian B, Glick H and Garland G (1994) Cholesterol and coronary heart disease: predicting risks by levels and ratios. *Ann Intern Med* **121**: 641–647

Love RR, Newcomb PA, Wiebe DA, Surawicz TS, Jordan VC, Carbone PP and DeMets DL (1990) Effects of tamoxifen therapy on lipid and lipoprotein levels in postmenopausal patients with node-negative breast cancer. *J Natl Cancer Inst* **82**: 1327–1332

Love RR, Cameron L, Connell BL and Leventhal H (1991) Symptoms associated with tamoxifen treatment in postmenopausal women. *Arch Intern Med* **151**: 1842–1847

Lukac J, Kusic Z, Kordic D, Koncar M and Bolanca A (1994) Natural killer cell activity, phagocytosis, and number of peripheral blood cells in breast cancer patients treated with tamoxifen. *Breast Cancer Res Treat* **29**: 279–285

McDonald CC, Alexander FE, White W, Forrest AP and Stewart HJ (1995) Cardiac and vascular morbidity in women receiving adjuvant tamoxifen for breast cancer in a randomized trial. *Br Med J* **311**: 977–980

Nachtigall LE (1995) Emerging delivery systems for estrogen replacement: aspects of transdermal and oral delivery. *Am J Obstet Gynecol* **173**: 993–997

Nayfield S (1995) Tamoxifen's role in chemoprevention of breast cancer. An update. *J Cell Biochem* suppl. 22: 42–50

Newcomb PA and Storer BE (1995) Postmenopausal hormone use and risk of large bowel cancer. *J Natl Cancer Inst* **87**: 1067–1071

Powles TJ, Hickish T, Casey S and O'Brien M (1993) Hormone replacement after breast cancer. *Lancet* **342**: 60–61

Powles TJ, Jones AL, Ashley SE, O'Brien ME, Tidy VA, Treleavan J, Cosgrove D, Nash AG, Sacks N, Baum M, McKinna JA and Davey JB (1994) The Royal Marsden Hospital pilot tamoxifen chemoprevention trial. *Breast Cancer Res Treat* **31**: 73–82

Powles TJ, Hickish T, Kanis JA, Tidy A and Ashley S (1996) Effect of tamoxifen on bone mineral density measured by dual-energy X-ray absorpiometry in healthy premenopausal and postmenopausal women. *J Clin Oncol* **14**: 78–84

Riggs B and Melton L (1992) The prevention of osteoporosis. *N Engl J Med* **327**: 620–627

Rosemberg L, Hennekens CHE, Rosner B, Belanger C, Rothman KJ and Speizer FE (1981) Early menopause and the risk of myocardial infarction. *Am J Obstet Gynecol* **139**: 47–51

Roy JA, Sawka CA and Pritchard KI (1996) Hormone replacement therapy in women with breast cancer. Do the risks outweigh the benefits? *J Clin Oncol* **14**: 997–1006

Rutqvist LE, Mattson A for the Stockholm Breast Cancer Study Group (1993) Cardiac and thromboembolic morbidity among postmenopausal women with early-stage breast cancer in a randomized trial of adjuvant tamoxifen. *J Natl Cancer Inst* **85**: 1398–1406

Rutqvist LE, Johansson H, Signomklao T, Johansson U, Fornander T and Wilking N (1995) Adjuvant tamoxifen therapy for early stage breast cancer and second primary malignancies. Stockholm Breast Cancer Study Group. *J Natl Cancer Inst* **87**: 645–651

Slowinska-Srzednicka J, Zgliczynski S, Jeske W, Stopinska-Gluszak U, Srzednicki M, Brzezinska A, Zgliczynski W and Sadowski Z (1992). Transdermal 17 beta-estradiol combined with oral progestagen increases plasma levels of insulin-like growth factor-I in postmenopausal women. *J Endocrinol Invest* **15**: 533–538

Smigel K (1998) Breast cancer prevention trial shows major benefit, some risk. *J Natl Cancer Inst* **90**: 647–648

Smith GD, Song F and Sheldon TA (1993) Cholesterol lowering and mortality: the importance of considering initial level of risk. *Br Med J* **306**: 1367–1373

Thaulow E, Erikssen J, Sandvik L, Stormorken H and Cohn PF (1991) Blood platelet count and function are related to total and cardiovascular death in apparently healthy men. *Circulation* **84**: 613–617

The Writing Group for the PEPI Trial (1995) Effects of estrogen or estrogen/progestin regimens on heart disease risk factors in postmenopausal women. The Postmenopausal Estrogen/Progestin Interventions (PEPI) Trial. *JAMA* **273**: 199–208

© Cancer Research Campaign 1998

Van Erpecum KJ, Van Berge Hengouwen GP, Verschoor GP, Verschoor L, Stoelwinder B and Willekens FLH (1991) Different hepatobiliary effects of oral and transdermal estradiol in postmenopausal women. *Gastroenterology* **100**: 482–488

Walsh BW, Li H and Sacks FM (1994) Effects of postmenopausal hormone replacement with oral and transdermal estrogen on high density lipoprotein metabolism. *J Lipid Res* **35**: 2083–2093

Weissberger AJ, Ho KK and Lazarus L (1991) Contrasting effects of oral and transdermal routes of estrogen replacement therapy on 24-hour growth hormone (GH) secretion, insulin-like growth factor-I, and GH-binding protein in postmenopausal women. *J Clin Endocrinol Metab* **72**: 374–381

Yang NN, Venugopalan M, Hardikar S and Glasebrook A (1996) Identification of an estrogen response element activated by metabolites of 17 beta-estradiol and raloxifene. *Science* **273**: 1222–1225, and comments in Pennisi E (1996) Drug's link to genes reveals estrogen's many sides. *Science* **273**: 1171

© Cancer Research Campaign 1998